# NT-proBNP as an Independent Predictor of Long-Term All-Cause Mortality in Heart Failure Across the Spectrum of Glomerular Filtration Rate

**DOI:** 10.3390/jcm14113886

**Published:** 2025-05-31

**Authors:** Anca Breha, Caterina Delcea, Andreea Cristina Ivanescu, Gheorghe-Andrei Dan

**Affiliations:** 1 Cardiology Department, Carol Davila University of Medicine and Pharmacy, 020021 Bucharest, Romania; anca.breha@drd.umfcd.ro (A.B.); andreea-cristina.ivanescu@umfcd.ro (A.C.I.);; 2 Cardiology Department, Colentina Hospital, 020125 Bucharest, Romania; 3 Academy of Romanian Scientists, 050044 Bucharest, Romania

**Keywords:** heart failure, NT-proBNP, chronic kidney disease, mortality, prognosis

## Abstract

**Background/Objectives:** The coexistence of heart failure (HF) and chronic kidney disease (CKD) complicates management and worsens prognosis. NT-proBNP is a recognized biomarker for HF diagnosis and prognosis, yet its interpretation in CKD can be challenging due to confounding factors increasing its levels. This study aimed to evaluate the predictive value of NT-proBNP for all-cause long-term mortality in HF patients across various stages of renal dysfunction. **Methods**: Hospitalized HF patients were included in this observational, retrospective analysis. NT-proBNP levels and serum creatinine were measured on admission. The primary outcome was all-cause mortality. Patients were divided into three groups according to renal function estimated using the CKD-EPI formula: eGFR1 (>60 mL/min/1.73 m^2^), eGFR2 (30–60 mL/min/1.73 m^2^) and eGFR3 (<30 mL/min/1.73 m^2^). **Results**: The study included 716 HF patients with a mean age of 71 ± 10 years, 49% males. All-cause long-term mortality was 35% after a median follow-up of 59 months. The mortality rate increased from 29% in eGFR1 patients, to 43% in eGFR2, to 68% in eGFR3. Median NT-proBNP increased from 997 pg/mL in eGFR1 patients to 1586 pg/mL in eGFR2 to 4928 pg/mL in eGFR3. Cut-off values for predicting all-cause long-term mortality were NT-proBNP >1837 pg/mL in eGFR1 patients, >1413 pg/mL in eGFR2 and >6415 pg/mL in eGFR3. In multivariable Cox analysis, NT-proBNP was an independent predictor of all-cause long-term mortality in all eGFR groups. **Conclusions**: NT-proBNP on admission was an independent predictor of long-term all-cause mortality in hospitalized HF patients across all eGFR subgroups, with increasing cut-off levels in patients with renal dysfunction.

## 1. Introduction

The intricate interplay of heart failure (HF) and kidney disease is reflected in the challenging management as well as the poor prognosis, when the two coexist [1,2]. Pathophysiological mechanisms involve hemodynamic, neurohormonal and disease-specific pathways that lead to accelerated decline in both cardiac as well as renal function [1]. The concurrence and bidirectional impact of the two diseases are associated with increased morbidity and mortality [3]. Therefore, early diagnosis, assessment and treatment are of utmost importance in order to improve the clinical course of these patients.

The brain natriuretic peptide (BNP) and its amino-terminal fragment (NT-proBNP) are established markers of both diagnosis and prognosis in HF [4]. NT-proBNP levels can be influenced by cardiac and non-cardiac factors such as atrial fibrillation (AF), aging, infections and KD, which may reduce their diagnostic specificity [5]. Thus, interpretation of NT-proBNP values may turn into a clinical dilemma in both acute and chronic kidney disease (CKD) and especially in end-stage renal disease (ESRD) [6,7,8]. Elevated NT-proBNP levels signal cardiomyocyte stretch; however, kidney dysfunction also independently drives NT-proBNP accumulation, often exceeding diagnostic thresholds. Despite these complexities, emerging evidence suggests that NT-proBNP remains a powerful predictor of cardiovascular morbidity and mortality in CKD and ESRD [7].

NT-proBNP is predominantly excreted by the kidneys [9]. As renal function declines, NT-proBNP levels rise exponentially, independently of cardiac performance, complicating its diagnostic utility. For every 10 mL/min/1.73 m^2^ decrease in estimated glomerular filtration rate (eGFR), BNP likely increases by 21%, and NT-proBNP by 38% [8,10,11]. However, this increase is not solely due to impaired clearance; worsening cardiac preload and afterload also drive peptide secretion, reinforcing NT-proBNP’s prognostic prediction [6,12].

Understanding these intricate dynamics is essential for refining cut-off values and optimizing cardiovascular risk assessment in patients with both HF and kidney dysfunction [13]. There is a lack of consensus on the appropriate NT-proBNP cut-off values for different levels of glomerular filtration rate (GFR). Previous research evaluated the diagnostic and prognostic role of NT-proBNP in patients with HF and CKD [14,15,16,17,18,19,20,21,22]; however, scarce data are available regarding a stratified approach to the use of natriuretic peptides in relation to the stages of kidney dysfunction, especially in patients with severe renal disease. There is strong evidence on the proportional increase in BNP and NT-proBNP with the decrease in GFR, and increased cut-off levels for survival prediction were proposed for patients with eGFR < 60 mL/min/1.73 m^2^ [16,17]. Research is still needed to evaluate NT-proBNP’s role and optimal cut-off values in relation to long-term mortality across the CKD spectrum. This aspect remains insufficiently explored, particularly regarding how renal dysfunction modifies the predictive value of NT-proBNP.

We therefore aimed to assess the independent predictive value and cut-off levels of NT-proBNP for all-cause long-term mortality across different ranges of glomerular filtration rate, in HF patients with and without renal dysfunction.

## 2. Materials and Methods

This is an observational, retrospective, single-center cohort study from a university hospital in Romania.

### 2.1. Study Population

All adult patients aged 18 years and older with HF admitted consecutively to the Cardiology Department between June 2018 and March 2020 were evaluated for inclusion. In-hospital mortality, readmissions of the same patient and pregnancy were exclusion criteria. All patients with NT-proBNP and creatinine levels measured within the first 2 h of admission and an echocardiographic evaluation during the index hospitalization were included. Patients who did not have NT-proBNP and creatinine levels measured within the first 2 h of admission, or an echocardiographic evaluation, were excluded.

### 2.2. Data Collection

Each patient’s dataset was obtained from the electronic medical records, and it included demographic details, comorbidities, clinical findings, laboratory results and medication history. Heart failure characteristics included the signs and symptoms of congestion, NYHA class, echocardiographic measurements and natriuretic peptide levels.

Results of the laboratory tests assessed on admission were recorded. NT-proBNP levels were assessed using a Roche Elecsys Cobas E801^®^ diagnostics assay. Blood samples were obtained within two hours of admission and were processed within a maximum of one hour.

### 2.3. Definitions

HF was diagnosed according to the European Society of Cardiology guidelines in patients with specific signs and symptoms, in the presence of structural and/or functional cardiac abnormalities resulting in elevated intracardiac pressures and/or inadequate cardiac output at rest and/or during exercise [23]. Furthermore, according to left ventricular ejection fraction (LVEF), patients were considered to have HF with preserved EF (HFpEF) if they had LVEF ≥ 50% with symptoms/signs of HF and evidence of cardiac structural/functional abnormalities or elevated natriuretic peptides, HF with mildly reduced EF (HFmrEF) if LVEF was between 41% and 49%, and HF with reduced EF (HFrEF) if they had LVEF ≤ 40% [23].

CKD was defined as kidney damage or glomerular filtration rate (GFR) < 60 mL/min/1.73 m^2^ for 3 months or more, irrespective of cause [24,25].

The CKD-EPI creatinine equation was employed to estimate GFR in mL/min/1.73 m^2^ body surface area using the serum creatinine (Scr) measured in mg/dL, as recommended by the KDIGO guidelines [25,26].

For the purpose of our study, we divided the cohort into three groups based on GFR: eGFR1 (eGFR > 60 mL/min/1.73 m^2^), eGFR2 (eGFR between 30–60 mL/min/1.73 m^2^) and eGFR3 (eGFR < 30 mL/min/1.73 m^2^).

### 2.4. Statistical Analysis

Statistical analysis was performed using Epi Info, 7.2.6.0 SPSS and MedCalc 22.009 software. Numerical variables with Gaussian distribution were expressed as mean ± standard deviation and were evaluated using the ANOVA test. Those with non-Gaussian distribution were expressed as median [interquartile range] and were evaluated using the Kruskal–Wallis test.

The Chi-square test was utilized to assess the relationship between qualitative variables, with the Yates correction applied where appropriate. Risk ratios were calculated for the analysis of groups with at least 50 patients achieving the outcome, and odds ratios otherwise. Receiver operating characteristic (ROC) curves were generated to determine the prognostic performance of predictors, with the Youden index applied to identify the optimal cut-off points.

Survival analysis was conducted using the Kaplan–Meier method to estimate survival curves. The Cox proportional hazards model was employed to evaluate the impact of various predictors on survival outcomes. For this purpose, variables significantly associated with the outcome identified in the univariable analysis were used. The forward conditional approach was employed to obtain the independent variables. After identifying the independent variables correlated with the outcome, we conducted another analysis by adding the NT-proBNP, in order to prove its independent predictive power.

Due to high inhomogeneity and non-Gaussian distribution of the NT-proBNP values, the logarithmic transformation in the base of 10 (Log_10_NT-proBNP) was used in the multivariable analysis.

Statistical significance was considered for a *p* value < 0.05.

## 3. Results

### 3.1. General Characteristics

The cohort included 716 HF patients with a mean age of 71 ± 10 years, 49% of whom were males. Two-thirds of patients had HFpEF and one-third had dyspnea at rest or on mild exertion. Of the total, 42% had ischemic etiology of HF and 59% had AF. The most prevalent risk factors were hypertension and dyslipidemia. One-third of the cohort had an eGFR < 60 mL/min/1.73 m^2^. The detailed characteristics of the study population are provided in Table 1.

All-cause long-term mortality was 35% after a median follow-up of 59 (38–66) months. Deceased patients were older, with a lower LVEF, a higher NYHA class and worse renal function. They had a significantly higher prevalence of AF, diabetes mellitus (DM) and history of stroke. Hypertension and dyslipidemia were less frequent among them (Table 1). They had higher NT-proBNP levels, and lower values of the eGFR, hemoglobin, serum sodium and total cholesterol (Table 1).

Across the eGFR groups, patients with lower kidney function were older, with a higher prevalence of atrial fibrillation and history of myocardial infarction. They also had lower sodium serum levels, higher serum potassium and blood glucose values and higher estimated pulmonary artery systolic pressure (Supplementary Table S1).

**Figure 1:**
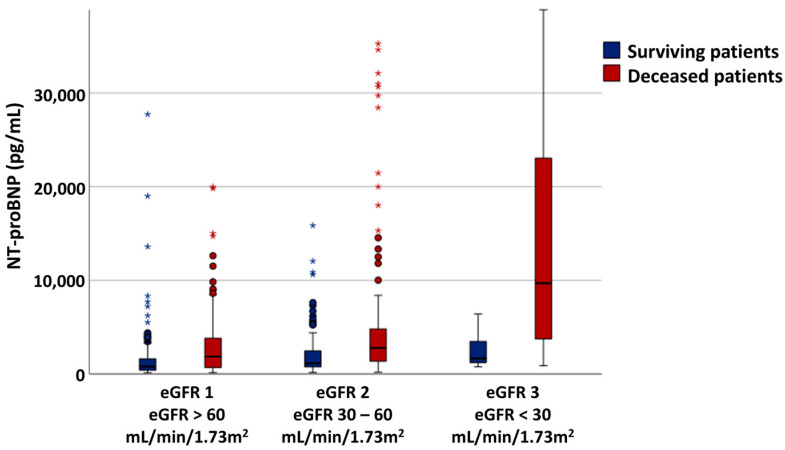
NT-proBNP variability across the eGFR ranges. * and circles are outliers.

NT-proBNP values increased proportionally with worsening renal function, and within each eGFR category. The surviving patients had significantly lower NT-proBNP values compared to the deceased ones, in each of the evaluated eGFR subgroups (Figure 1, Table 2).

### 3.2. Univariable Survival Analysis Stratified by the Renal Function

Patients with an eGFR greater than 60 mL/min/1.73 m^2^ had a mortality rate of 28.66%. Clinical parameters associated with the outcome were age, male sex, higher NYHA class, AF, history of stroke, infection, malignancy and cirrhosis (Table 3). Laboratory and echocardiography parameters associated with the primary outcome were hemoglobin levels, absolute number of neutrophils, renal function, AST and total cholesterol, LVEF and estimated pulmonary artery systolic pressure (PASP) (Table 3). In this subgroup, NT-proBNP was a significant predictor of all-cause mortality, with a cut-off value of >1837 pg/mL (Table 4).

Patients with eGFR between 60 and 30 mL/min/1.73 m^2^ had a mortality rate of 43.13%. Age, higher NYHA class, AF, infection, malignancy, hemoglobin, serum sodium levels, LVEF and PASP were associated with the outcome (Table 3). In this subgroup, NT-proBNP was a significant predictor of all-cause mortality, with a cut-off value of >1413 pg/mL (Table 4).

Patients with eGFR below 30 mL/min/1.73 m^2^ had a mortality rate of 67.65%. Hemoglobin levels, platelet count, serum potassium and PASP were associated with all-cause mortality (Table 3). NT-proBNP was a significant predictor of all-cause mortality, with a cut-off value of > 6415 pg/mL (Table 4).

**Figure 2:**
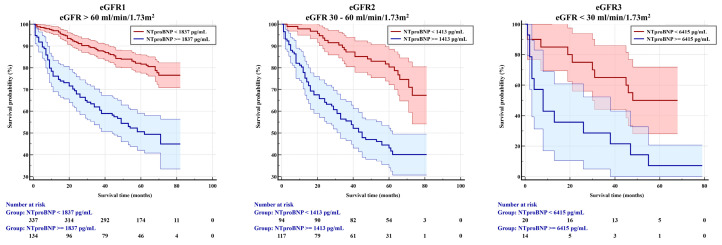
Kaplan–Meier survival analysis (patients at risk).

In Kaplan–Meier survival analysis using the NT-proBNP cut-off levels previously determined, lower eGFR was associated with lower survival time, which was significantly decreasing among the renal function subgroups from 51 months in patients with eGFR1 to 46 months in those with eGFR2 to 21 months in those with eGFR3 (Table 5, Figure 2).

### 3.3. Multivariable Survival Analysis

In Cox analysis, the independent predictors of all-cause long-term mortality in patients with eGFR > 60 mL/min/1.73 m^2^ were male sex, NYHA class 3 or 4, malignancy, hemoglobin levels, neutrophil count and PASP (Table 6). NT-proBNP evaluated as a continuous variable as Log_10_NT-proBNP was an independent predictor of the outcome.

For patients with eGFR between 60 and 30 mL/min/1.73 m^2^, the independent mortality predictors in the initial analysis were age, hemoglobin levels, PASP, LVEF and serum sodium. NT-proBNP evaluated as a continuous variable as Log10NT-proBNP was an independent predictor of the outcome, and outperformed LVEF and age which lost their predictive power (Table 6).

Patients with eGFR < 30 mL/min/1.73 m^2^ had two independent predictors of all-cause long-term mortality in the initial Cox analysis: PASP and total cholesterol. When NT-proBNP was added to the model, total cholesterol lost its predictive power. NT-proBNP, evaluated as a continuous variable as Log_10_NT-proBNP, was an independent predictor of the outcome, and outperformed LVEF and age, which lost their predictive power.

## 4. Discussion

Our analysis of this cohort of hospitalized HF patients with or without renal impairment showed that NT-proBNP values increased with the decrease in eGFR; however, it remained an independent predictor of all-cause long-term mortality across the eGFR spectrum, with higher cut-off levels in patients with more advanced kidney dysfunction. A steep increase in the cut-off point was observed in patients with eGFR < 30 mL/min, where the obtained cut-off value for mortality prediction was 6415 pg/mL. Moreover, within the decreasing eGFR across the studied subgroups, fewer clinical or non-clinical parameters remained independently associated with the outcome.

### 4.1. NT-proBNP Levels Across GFR Subgroups

Our results are consistent with previous data confirming the rising values of natriuretic peptides in progressively higher stages of kidney dysfunction. Relatedly, cardiac troponin levels, reflecting myocardial damage as well as comorbidity burden resulting in cardiac injury, were proven to be independent predictors of survival and rehospitalization in HF patients with kidney disease, in a recent systematic review [27].

NT-proBNP concentrations have been shown to correlate inversely with GFR in patients with renal impairment. In a cohort of 177 non-diabetic patients with mild-to-moderate CKD, NT-proBNP levels increased proportionally to decreasing eGFR, and the cut-off value of 213 ng/L was predictive for CKD progression [19]. Higher median NT-proBNP levels were also reported by Fandini et al. in patients with CKD compared to those without (238.5 pg/mL vs. 44.0 pg/mL; *p* < 0.001) [28]. An analysis of the CRIC and SPRINT cohorts including patients without previously diagnosed HF revealed increasing values of the 95th percentile of NT-proBNP levels from 682 pg/mL in subjects with eGFR 45–59 mL/min/1.73 m^2^ to 1130 pg/mL for those with eGFR 30–44 mL/min/1.73 m^2^ to 2523 pg/mL in those with eGFR < 30 mL/min/1.73 m^2^ [29].

The consistent elevation in NT-proBNP levels may be partially attributed to the nadir of the renal function’s inability to clear this peptide from circulation, further enhancing its reliability as a prognostic marker. Moreover, for patients with cardiovascular disease and an eGFR < 30 mL/min/1.73 m^2^, the optimal cut-off for predicting all-cause death and major cardiovascular events was found to be markedly higher, at 5809.0 pg/mL, as compared to 258.6 pg/mL for those with an eGFR ≥ 30 mL/min/1.73 m^2^ [30].

In line with our findings, another systematic review and meta-analysis looking into the clinical utility of NT-proBNP for acute decompensated HF proved the biomarker’s preserved diagnostic and prognostic value in patients with renal dysfunction, with higher values than the normal population [31]. In a cohort of 341 patients with stable CHF, the cut-off levels for NT-proBNP for predicting cardiac events or hospitalization due to worsening HF were ≥ 1474 pg/mL for those with eGFR < 60 mL/min [17], similar to the cut-off found in our study sample for all-cause mortality in patients with the same GFR. In Chinese patients with coronary artery disease followed-up for 417 days for all-cause mortality, different cut-off values were found for those with and without CKD [32]. In this cohort, for those with eGFR < 60 mL/min, NT-proBNP > 370 pg/mL was the threshold for worse prognosis, while for those with eGFR ≥ 60 mL/min, the limit was much higher, with an NT-proBNP > 2584 pg/mL [32]. Confirming our results for patients with eGFR < 30mL/min, Horii et al. found that NT-proBNP > 5809 pg/mL was significantly associated with all-cause mortality [30].

We therefore advocate for the clinical use of NT-proBNP as a predictor of all-cause long-term mortality in patients with HF regardless of kidney function, given that it maintained its prognostic value across the eGFR subgroups, with inversely proportionally increasing values to the decline in renal function.

### 4.2. Multivariable Mortality Prediction Across eGFR Subgroups

This study certified that NT-proBNP, a primary marker of myocardial dysfunction and degree of renal damage, and PASP, an indicator of increased LV filling pressures and pulmonary hypertension (PH), were the two persistent independent predictors of all-cause long-term mortality across all the eGFR subgroups. Previous data showed that in patients with CKD, PH incidence was determined by age, anemia, decreased LVEF and left ventricular hypertrophy [33]. Concordant with our results, increasing PASP and the presence of PH were previously independently associated with a higher risk of death in CKD patients [33], as well as in end-stage renal disease patients [34], reinforcing pulmonary hypertension as a key risk factor for mortality in this patient population.

A multitude of novel biomarkers have been assessed with the purpose of improving HF prognosis [35,36,37], alongside multiparametric risk models that aimed at increasing the predictive accuracy [38,39]. In a recent comparison, the BCN-Bio-HF score, which included NT-proBNP values, besides renal function, clinical variables and other laboratory parameters, had the best analytical performance compared with other prognostic scores, highlighting the biomarker’s prognostic value [40].

A novel approach of our study was the distinct analysis for patients from separate eGFR groups, showing different independent parameters for those with progressively worse kidney function. Our results underline the importance of HF severity, alongside the severity of renal dysfunction for the patients with eGFR < 30 mL/min/1.73m^2^. For these patients, other commonly used predictors lost their independent prognostic value. For example, in patients with eGFR > 90 mL/min or with eGFR between 30–60 mL/min, hemoglobin levels were independently associated with mortality, as previously proven in other cohorts [41], while in patients with eGFR < 30 mL/min, it was no longer correlated with the outcome in the multivariable analysis. The triad of HF, CKD and anemia is known to significantly impact survival prognosis [18]; however, we may argue that its predictive role is less reliable for advanced heart and kidney disease, where the two pathologies are the main drivers of mortality risk. Concordant results were reported for patients with acute cardiorenal syndrome, having the kidney function on admission as a key predictor of prognosis [42].

### 4.3. Limitations

The primary limitations of our study are its retrospective design and the fact that it was conducted at a single center. While this allowed us to include a larger number of patients and to have a follow-up period of over 4 years, it restricted the number of variables that could be assessed (including but not limited to the data regarding previous cardiac surgery). However, we consider that for the purpose of identifying the utility and cut-off values of NT-proBNP across different eGFRs for predicting all-cause mortality, we were able to include in the multivariable analysis the main cardiovascular and non-cardiovascular factors that could represent potential confounders.

The subgroup of patients with eGFR below 30 mL/min/1.73 m^2^ was rather small, influencing our findings’ strength and reliability. However, we argue that the obtained cut-off for NT-proBNP in relation to the primary endpoint was similar to that previously reported from a different cohort [30]. Moreover, the statistically significant parameters that were associated with the outcome in this subgroup had a *p* value < 0.01 in univariable regression, reinforcing their validity.

Our primary endpoint was all-cause mortality. We acknowledge that the lack of data on cardiovascular mortality is a limitation of our study. However, we argue that all-cause mortality is a strong primary endpoint used in many HF trials, more robust and less prone to uncertainty and bias [43,44].

Our univariable and multivariable analyses were confined to the readily available clinical, echocardiographic and laboratory parameters. While experimental studies included novel biomarkers of cardiac or renal function, since they are not routinely used in clinical practice, we argue that our results prove the use of NT-proBNP in regular clinical practice across the eGFR range.

## 5. Conclusions

The results of our study confirm that in hospitalized HF patients, NT-proBNP values on admission are predictive for all-cause long-term mortality, across all groups of eGFR ranging from normal to severe renal dysfunction. While cut-off levels for the outcome ranged from NT-proBNP > 1413 to 1991 pg/mL for our general cohort and for eGFR groups > 30 mL/min, the highest NT-proBNP cut-off value > 6415 pg/mL was predictive of all-cause mortality in patients with eGFR < 30 mL/min.

## Figures and Tables

**Table 1:** General clinical and laboratory characteristics of the study cohort on admission.

		All Patients (N = 716)	Surviving Patients (N = 465)	Deceased Patients (N = 251)	*p*-Value
General characteristics					
Age (years)		71 ± 10.04	69 ± 9.66	75 ± 9.8	<0.0001
Gender (male)		348 (48.6%)	214 (46%)	134 (53.3%)	0.06
Clinical characteristics					
Heart rate (bpm)		79.85 ± 22.86	79.38 ± 22.48	80.66 ± 23.74	0.48
Systolic blood pressure (mmHg)		136.23 ± 24.04	137.38 ± 23.58	134.18 ± 24.98	0.09
Diastolic blood pressure (mmHg)		79.33 ± 12.17	80.35 ± 12.03	77.29 ± 12.34	<0.001
Heart failure characteristics					
LVEF (%)		46.5 ± 13.3	48.06 ± 12.28	43.8 ± 14.73	<0.0001
HFpEF		420 (58.65%)	279 (60%)	141 (56.1%)	0.1
HFmrEF		131 (18.29%)	90 (19.35%)	41 (16.33%)	
HFrEF		165 (23%)	96 (20.6%)	69 (27.49%)	
NYHA I		40 (5.58%)	28 (6.02%)	12 (4.78%)	<0.0001
NYHA II		488 (68.1%)	350 (75.2%)	138 (54.98%)	0.002
NYHA III		170 (23.74%)	83 (17.84%)	87 (34.66%)	<0.0001
NYHA IV		18 (2.51%)	4 (0.86%)	14 (5.57%)	0.04
Renal function characteristics					
Creatinine (mg/dL)		1.05 (0.66)	0.96 (0.33)	1.22 (1.00)	<0.0001
eGFR (ml/min/1.73 m^2^)		69.6 (22.7)	73.62 (21.47)	62.19 (23.15)	<0.0001
eGFR > 60 mL/min/1.73 m^2^		83.02 ± 13.79	84.15 ± 14.29	80.02 ± 11.99	0.003
eGFR 30–60 mL/min/1.73 m^2^		47.86 ± 7.74	48.46 ± 7.47	46.93 ± 8.02	0.15
eGFR < 30 mL/min/1.73 m^2^		21.93 ± 6.15	24.88 ± 5.04	20.52 ± 6.23	0.05
Uric acid (mg/dL)		6.33 ± 1.86	6.1 ± 1.67	6.77 ± 2.1	<0.0001
Risk factors and comorbidities					
Ischemic heart disease		302 (42.17%)	206 (44.3%)	96 (38.2%)	0.11
Prior myocardial infarction		130 (18.15%)	78 (16.77%)	52 (20.7%)	0.19
Stable angina		121 (16.89%)	87 (18.7%)	34 (13.5%)	0.07
HTN		621 (86.7%)	412 (88.6%)	209 (83.2%)	0.037
Diabetes mellitus		250 (34.9%)	149 (32%)	101 (40.2%)	0.028
Dyslipidemia		550 (76.8%)	376 (80%)	174 (69.3%)	<0.0001
History of stroke/TIA		96 (13.4%)	51 (10.96%)	45 (17.9%)	0.009
AF		419 (58.5%)	249 (53.5%)	170 (67.7%)	<0.0001
Type of AF	Paroxysmal	91 (12.7%)	55 (11.82%)	36 (14.34%)	<0.001
	Persistent	108 (15%)	74 (15.9%)	34 (13.54%)	0.38
	Permanent	218 (30.4%)	120 (25.8%)	98 (39%)	0.02
Peripheral arterial disease		67 (9.35%)	43 (9.24%)	24 (9.56%)	0.89
Obesity		265 (37%)	184 (39.5%)	81 (32.2%)	0.054
COPD		40 (5.58%)	22 (4.73%)	18 (7.17%)	0.178
Laboratory parameters					
Serum sodium (mmol/L)		140.55 ± 3.47	140.9 ± 2.79	139.78 ± 4.3	<0.0001
Serum potassium (mmol/L)		4.45 ± 0.5	4.46 ± 0.5	4.44 ± 0.51	0.71
Serum chloride (mmol/L)		101.28 ± 5.43	101.69 ± 5.76	100.51 ± 4.77	0.03
Blood glucose (mg/dL)		118 ± 40	116.9 ± 37.5	120.6 ± 44.7	0.24
Total cholesterol (mg/dL)		165 ± 47.74	171.68 ± 47.9	153.45 ± 45.2	<0.0001
AST (UI/L)		19.5 [14.4–30]	19.3 [14.5–31.2]	19.1 [14.1–27]	0.43
ALT (UI/L)		21.8 [17.8–28.5]	21.5 [17.7–27.5]	23.1 [18.2–31.1]	0.66
NT-proBNP (pg/mL)		1187 [580–2713]	967 [485–1796]	2398 [1091–5084]	<0.0001

AF: atrial fibrillation, ALT: alanine aminotransferase, AST: aspartate aminotransferase, bpm: beats per minute, COPD: chronic obstructive pulmonary disease, eGFR: estimated glomerular filtration rate, HFpEF: heart failure with preserved ejection fraction, HFmEF: heart failure with mildly reduced ejection fraction, HFrEF: heart failure with reduced ejection fraction, LVEF: left ventricular ejection fraction, HTN: hypertension, NYHA: New York Heart Association, TIA: transient ischemic attack.

**Table 2:** NT-proBNP variability within eGFR subgroups.

	eGFR1 eGFR > 60 mL/min/1.73 m^2^		eGFR2 eGFR 30–60 mL/min/1.73 m^2^		eGFR3 eGFR < 30 mL/min/1.73 m^2^	
NT-proBNP (pg/mL)	997 [461–2110]		1586 [871–3473]		4928.5 [2030–17,464]	
	*p* value for trend * < 0.001					
	Surviving patients	Deceased patients	Surviving patients	Deceased patients	Surviving patients	Deceased patients
NT-proBNP (pg/mL)	799.4 [404–1624]	1850 [685.2–3818]	1135 [751–2472]	2795 [1281–4879]	1667 [1163–3497]	9690 [3143–23,738]
*p* value **	<0.001		<0.001		<0.001	

* comparison between eGFR subgroups; ** comparison between surviving and deceased patients; eGFR, estimated glomerular filtration rate.

**Table 3:** Univariable analysis of predictors for all-cause mortality.

	eGFR1 eGFR > 60 mL/min/1.73 m^2^ N = 471	eGRF2 eGFR 30–60 mL/min/1.73 m^2^ N = 211	eGFR3 eGFR < 30 mL/min/1.73 m^2^ N = 34
	Clinical characteristics and comorbidities		
	AUC, 95% CI *p* value		
Age	0.637, 0.581–0.693 *p* < 0.001	0.655, 0.576–0.733 *p* < 0.001	0.470, 0.255–0.685 *p* = 0.78
	RR, 95% CI *p* value		
Male sex	1.19, 1.06–1.34 *p* = 0.005	1.10, 0.86–1.41 *p* = 0.51	0.77, 0.17–3.48 *p* = 0.73
NYHA 3/4	1.42, 1.17–1.71 *p* < 0.001	1.67, 1.21–2.31 *p* < 0.001	2.91, 0.61–13.83 *p* = 0.32
IHD	0.99, 0.88–1.12 *p* = 1.00	0.79, 0.63–1.101 *p* = 0.08	0.07, 0.01–0.59 *p* = 0.01
Prior MI	1.15, 0.95–1.39 *p* = 0.12	0.99, 0.74–1.33 *p* = 0.96	0.23, 0.05–1.08 *p* = 0.13
AF	1.124, 1.02–1.28 *p* = 0.03	1.34, 1.07–1.69 *p* = 0.02	3.00, 0.64–14.08 *p* = 0.31
HTN	0.84, 0.69–1.03 *p* = 0.08	0.74, 0.44–1.23 *p* = 0.27	0.48, 0.50–4.84 *p* = 0.90
DM	1.11, 0.97–1.27 *p* = 0.10	1.06, 0.82–1.36 *p* = 0.78	1.91, 0.44–8.35 *p* = 0.62
History of stroke	126, 1.01–1.58 *p* = 0.03	1.02, 0.71–1.46 *p* = 0.92	5.33, 0.57–49.48 *p* = 0.24
COPD	1.18, 0.82–1.70 *p* = 0.40	1.23, 0.70–2.13 *p* = 0.60	0.95, 0.08–11.79 *p* = 0.97
Thyroid disease	1.15, 0.88–1.49 *p* = 0.32	1.17, 0.75–1.80 *p* = 0.062	0.80, 0.04–17.19 *p* = 0.89
Infection	1.30, 1.00–1.70, *p* = 0.03	1.87, 1.01–3.48 *p* = 0.024	4.37, 0.47–41.07 *p* = 0.35
Malignancy	1.49, 1.09–2.06 *p* = 0.002	3.39, 0.96–11.99 *p* = 0.013	N/A
PE	1.06, 0.60–1.87 *p* = 1.00	1.23, 0.46–3.31 *p* = 0.64	N/A
Cirrhosis	2.14, 0.85–5.41 *p* = 0.03	N/A	N/A
	Laboratory parameters		
	AUC (95% CI), *p* value		
WBC *	0.510, 0.439–0.581 *p* = 0.78	0.434, 0.352–0.517 *p* = 0.12	0.712, 0.519–0.906 *p* = 0.06
Neutrophils	0.565, 0.504–0.627 *p* = 0.03	0.508, 0.428–0.587 *p* = 0.85	0.561, 0.356–0.766 *p* = 0.60
Hb *	0.672, 0.613–0.730 *p* < 0.001	0.678, 0.603–0.752 *p* < 0.001	0.737, 0.564–0.910 *p* = 0.04
PLT *	0.555, 0.495–0.615 *p* = 0.06	0.514, 0.432–0.596 *p* = 0.73	0.765, 0.604–0.927 *p* = 0.02
Blood glucose	0.533, 0.465–0.600 *p* = 0.34	0.449, 0.365–0.532 *p* = 0.225	0.623, 0.407–0.838 *p* = 0.27
Serum Na *	0.555, 0.494–0.616 *p* = 0.07	0.602, 0.524–0.681 *p* = 0.01	0.491, 0.289–0.692 *p* = 0.93
Serum K *	0.538, 0.479–0.597 *p* = 0.21	0.514, 0.433–0.594 *p* = 0.74	0.766, 0.570–0.961 *p* = 0.02
Serum Cl *	0.551, 0.491–0.611 *p* = 0.09	0.558, 0.479–0.638 *p* = 0.15	0.483, 0.273–0.692 *p* = 0.87
Creatinine	0.566, 0.510–0.622 *p* = 0.03	0.528, 0.443–0.612 *p* = 0.514	0.676, 0.486–0.866 *p* = 0.10
eGFR *	0.583, 0.528–0.638 *p* = 0.005	0.553, 0.474–0.632 *p* = 0.19	0.700, 0.509–0.890 *p* = 0.06
AST	0.582, 0.515–0.650 *p* = 0.02	0.472, 0.380–0.564 *p* = 0.55	0.561, 0.344–0.779 *p* = 0.59
ALT	0.488, 0.422–0.555 *p* = 0.74	0.465, 0.372–0.557 *p* = 0.45	0.628, 0.388–0.867 *p* = 0.27
Total cholesterol *	0.642, 0.578–0.707 *p* < 0.001	0.528, 0.432–0.625 *p* = 0.56	0.826, 0.685–0.967 *p* = 0.003
Echocardiography parameters			
AUC (95% CI), *p* value			
LVEF	0.567, 0.507–0.627 *p* = 0.03	0.584, 0.505–0.663 *p* = 0.04	0.511, 0.302–0.721 *p* = 0.92
PASP	0.698, 0.634–0.762 *p* < 0.001	0.700, 0.621–0.778 *p* < 0.001	0.759, 0.599–0.919 *p* = 0.016

AF: atrial fibrillation, ALT: alanine aminotransferase, AST: aspartate aminotransferase, COPD: chronic obstructive pulmonary disease, DM: diabetes mellitus, eGFR: estimated glomerular filtration rate, Hb: hemoglobin, K; potassium, LVEF: left ventricular ejection fraction, HTN: hypertension, Na: serum sodium, N/A: not applicable, NYHA: New York Heart Association, PASP: pulmonary artery systolic pressure, PE: pulmonary embolism, PLT: platelets, TIA: transient ischemic attack, WBC: white blood cells. * Lower values correlate with the outcome.

**Table 4:** ROC analysis and NT-proBNP cut-off values across the eGFR spectrum.

eGFR Category (mL/min/1.73 m^2^)	AUC (95% CI)	Cut-Off Value (pg/mL) Sensitivity, Specificity	*p* Value
All group	0.726, 0.692–0.759	>1991 55.78%, 78.91%	<0.001
eGFR1 eGFR > 60	0.684, 0.640–0.726	>1837 50.40%, 80.10%	<0.001
eGFR2 eGFR 30–60	0.717, 0.651–0.777	>1413 74.73%, 58.82%	<0.001
eGFR3 eGFR < 30	0.850, 0.686–0.949	>6415 58.33%, 100%	<0.001

AUC: area under the curve, CI: confidence interval, eGFR: estimated glomerular filtration rate.

**Table 5:** Kaplan–Meier survival analysis (time).

eGFR (mL/min/1.73 m^2^)	NT-proBNP Cut-Off Level (pg/mL)	Surviving Patients Follow-Up Time (Months)	Deceased Patients Survival Duration (Months)	Chi Square	Logrank *p* Value
>60	>1837	71.46 ± 1.22	50.92 ± 2.85	55.47	<0.001
30–60	>1413	69.81 ± 2.16	46.21 ± 2.96	29.69	<0.001
<30	>6415	46.45 ± 5.53	20.57 ± 6.39	8.99	0.003

**Table 6:** Multivariable Cox analysis for all-cause mortality.

	eGFR >60 mL/min/1.73 m^2^		eGFR 30–60 mL/min/1.73 m^2^		eGFR <30 mL/min/1.73 m^2^	
Step 1	Male sex	2.60, 1.56–4.35 *p* = 0.001	Age	1.06, 1.03–1.10 *p* < 0.001	PASP	1.04, 1.02–1.10 *p* = 0.042
	NYHA 3/4	2.28, 1.35–3.84 *p* = 0.002	Hb	0.85, 0.76–0.90 *p* = 0.004	TC	0.99, 0.98–1.00 *p* = 0.045
	Malignancy	2.05, 1.05–4.01 *p* = 0.036	PASP	1.03, 1.01–1.04 *p* = 0.002		
	Hb	0.81, 0.71–0.94 *p* = 0.004	LVEF	0.97, 0.95–0.99 *p* < 0.001		
	Neutrophils	1.00, 1.00–1.00 *p* < 0.001	Serum Na	0.93, 0.86–0.99 *p* = 0.036		
	PASP	1.03, 1.01–1.04 *p* < 0.001				
Step 2 = Step 1 + Log_10_BNP	PASP	1.03, 1.01–1.04 *p* < 0.001	PASP	1.02, 1.01–1.04 *p* = 0.005	PASP	1.06, 1.03–1.10 *p* = 0.02
	Hb	0.77, 0.69–0.86 *p* < 0.001	Hb	0.86, 0.77–0.96 *p* = 0.006	Log_10_BNP	2.53, 1.05–6.10 *p* = 0.04
	NYHA 3/4	1.92, 1.25–2.96 *p* = 0.003	Log_10_BNP	3.32, 1.96–5.63 *p* < 0.001		
	Sex	2.59, 1.69–3.95 *p* < 0.001				
	Neutrophils	1.00, 1.00–1.00				
	Malignancy	2.11, 1.20–3.72 *p* = 0.010				
	Log_10_BNP	1.87, 1.11–3.18 *p* = 0.020				
Variables without independent predictive value	Age, AF, stroke, infection, cirrhosis, GOT, total cholesterol, LVEF, eGFR		NYHA 3/4, Malignancy, AF, Infection		Serum potassium, Hemoglobin	

## Data Availability

The raw data supporting the conclusions of this article will be made available by the authors on request.

## Supplementary Materials

The following supporting information can be downloaded at: https://www.mdpi.com/article/10.3390/jcm14113886/s1, Table S1. General clinical and laboratory characteristics of the study cohort on admission, across the eGFR subgroups.

## Abbreviations

The following abbreviations are used in this manuscript:

AF	atrial fibrillation
ALT	alanine aminotransferase
AST	aspartate aminotransferase
AUC	area under the curve
COPD	chronic obstructive pulmonary eisease
CI	confidence interval
DM	diabetes mellitus
eGFR	estimated glomerular filtration rate
Hb	hemoglobin
HFpEF	heart failure with preserved ejection fraction
HFmEF	heart failure with mid-range ejection fraction
HFrEF	heart failure with reduced ejection fraction
K	potassium
LVEF	left ventricular ejection fraction
HTN	hypertension
NA	sodium
NYHA	York Heart Association
PASP	pulmonary artery systolic pressure
PE	pulmonary embolism
PLT	platelets
TIA	transient ischemic attack
WBC	white blood cells

## References

[1] Schefold J.C., Filippatos G., Hasenfuss G., Anker S.D., von Haehling S. (2016). Heart failure and kidney dysfunction: Epidemiology, mechanisms and management. Nat. Rev. Nephrol..

[2] Wang S., Li M., Wang X., Luo J., Zou Y., Hu Y., Liu X., Ao H., Yao X., Li C. (2021). The Ratio of NT-proBNP to CysC^1.53^ Predicts Heart Failure in Patients with Chronic Kidney Disease. Front. Cardiovasc. Med..

[3] Damman K., Valente M.A.E., Voors A.A., O’Connor C.M., Van Veldhuisen D.J., Hillege H.L. (2014). Renal impairment, worsening renal function, and outcome in patients with heart failure: An updated meta-analysis. Eur. Heart J..

[4] Niizuma S., Iwanaga Y., Yahata T., Miyazaki S. (2017). Renocardiovascular Biomarkers: From the Perspective of Managing Chronic Kidney Disease and Cardiovascular Disease. Front. Cardiovasc. Med..

[5] Mueller C., McDonald K., de Boer R.A., Maisel A., Cleland J.G.F., Kozhuharov N., Coats A.J.S., Metra M., Mebazaa A., Ruschitzka F. (2019). Heart Failure Association of the European Society of Cardiology practical guidance on the use of natriuretic peptide concentrations. Eur. J. Heart Fail..

[6] Harrison T.G., Shukalek C.B., Hemmelgarn B.R., Zarnke K.B., Ronksley P.E., Iragorri N., Graham M.M., James M.T. (2020). Association of NT-proBNP and BNP With Future Clinical Outcomes in Patients With ESKD: A Systematic Review and Meta-analysis. Am. J. Kidney Dis..

[7] Niizuma S., Iwanaga Y., Yahata T., Tamaki Y., Goto Y., Nakahama H., Miyazaki S. (2009). Impact of left ventricular end-diastolic wall stress on plasma B-type natriuretic peptide in heart failure with chronic kidney disease and end-stage renal disease. Clin. Chem..

[8] Takase H., Dohi Y. (2014). Kidney function crucially affects B-type natriuretic peptide (BNP), N-terminal proBNP and their relationship. Eur. J. Clin. Investig..

[9] Goetze J.P., Jensen G., Møller S., Bendtsen F., Rehfeld J.F., Henriksen J.H. (2006). BNP and N-terminal proBNP are both extracted in the normal kidney. Eur. J. Clin. Investig..

[10] McCullough P.A., Duc P., Omland T., McCord J., Nowak R.M., Hollander J.E., Herrmann H.C., Steg P.G., Westheim A., Knudsen C.W. (2003). B-type natriuretic peptide and renal function in the diagnosis of heart failure: An analysis from the Breathing Not Properly Multinational Study. Am. J. Kidney Dis..

[11] Jafri L., Kashif W., Tai J., Siddiqui I., Azam I., Shahzad H., Ghani F. (2013). B-type natriuretic peptide versus amino terminal pro-B type natriuretic peptide: Selecting the optimal heart failure marker in patients with impaired kidney function. BMC Nephrol..

[12] Yang W.L., Fahim M., Johnson D.W. (2020). Pathophysiology and significance of natriuretic peptides in patients with end-stage kidney disease. Clin. Biochem..

[13] van Kimmenade R.R., Januzzi J.L., Baggish A.L., Lainchbury J.G., Bayes-Genis A., Richards A.M., Pinto Y.M. (2006). Amino-Terminal Pro-Brain Natriuretic Peptide, Renal Function, and Outcomes in Acute Heart Failure. Redefining the Cardiorenal Interaction?. J. Am. Coll. Cardiol..

[14] Wu Z., Xu L., Cai A., Chen W., Xia S., He X., Qiu W., Xiao X., Gao Z., Chen J. (2022). The Prognostic Significance of Chronic Kidney Disease on the All-Cause Death of Ischemic Heart Failure in the Chinese Population: A Prospective Cohort Study. Cardiorenal Med..

[15] Linzbach S., Samigullin A., Yilmaz S., Tsioga M. (2009). Role of N-Terminal Pro-Brain Natriuretic Peptide and Cystatin C to Estimate Renal Function in Patients With and Without Heart Failure. Atlanta J..

[16] Palladini G., Foli A., Milani P., Russo P., Albertini R., Lavatelli F., Obici L., Perlini S., Moratti R., Merlini G. (2012). Best use of cardiac biomarkers in patients with AL amyloidosis and renal failure. Am. J. Hematol..

[17] Bruch C., Fischer C., Sindermann J., Stypmann J., Breithardt G., Gradaus R. (2008). Comparison of the Prognostic Usefulness of N-Terminal Pro-Brain Natriuretic Peptide in Patients With Heart Failure With Versus Without Chronic Kidney Disease. Am. J. Cardiol..

[18] Virani S.A., Khosla A., Levin A. (2008). Chronic kidney disease, heart failure and anemia. Can. J. Cardiol..

[19] Spanaus K.S., Kronenberg F., Ritz E., Schlapbach R., Fliser D., Hersberger M., Kollerits B., König P., von Eckardstein A., Mild-to-Moderate Kidney Disease Study Group (2007). B-type natriuretic peptide concentrations predict the progression of nondiabetic chronic kidney disease: The mild-to-moderate kidney disease study. Clin. Chem..

[20] Satyan S., Light R.P., Agarwal R. (2007). Relationships of N-Terminal Pro-B-Natriuretic Peptide and Cardiac Troponin T to Left Ventricular Mass and Function and Mortality in Asymptomatic Hemodialysis Patients. Am. J. Kidney Dis..

[21] Haapio M., Ronco C. (2008). BNP and a renal patient: Emphasis on the unique characteristics of B-type natriuretic peptide in end-stage kidney disease. Contrib. Nephrol..

[22] Han X., Zhang S., Chen Z., Adhikari B.K., Zhang Y., Zhang J., Sun J., Wang Y. (2020). Cardiac biomarkers of heart failure in chronic kidney disease. Clin. Chim. Acta.

[23] McDonagh T.A., Metra M., Adamo M., Gardner R.S., Baumbach A., Böhm M., Burri H., Butler J., Čelutkienė J., Chioncel O. (2021). 2021 ESC Guidelines for the diagnosis and treatment of acute and chronic heart failure. Eur. Heart J..

[24] Stevens P.E., Levin A. (2013). Evaluation and management of chronic kidney disease: Synopsis of the kidney disease: Improving global outcomes 2012 clinical practice guideline. Ann. Intern. Med..

[25] Kidney Disease: Improving Global Outcomes (KDIGO) CKD Work Group (2024). KDIGO 2024 Clinical Practice Guideline for the Evaluation and Management of Chronic Kidney Disease. Kidney Int..

[26] Levey A.S., Stevens L.A., Schmid C.H., Zhang Y.L., Castro A.F., Feldman H.I., Kusek J.W., Eggers P., Van Lente F., Greene T. (2009). A new equation to estimate glomerular filtration rate. Ann. Intern. Med..

[27] Breha A., Delcea C., Ivanescu A.C., Dan G.A. (2025). The Prognostic Value of Troponin Levels Adjusted for Renal Function in Heart Failure-A Systematic Review. Rom. J. Intern. Med..

[28] Fadini G.P., Bonora B.M., Albiero M., Zaninotto M., Plebani M., Avogaro A. (2017). DPP-4 inhibition has no acute effect on BNP and its N-terminal pro-hormone measured by commercial immune-assays. A randomized cross-over trial in patients with type 2 diabetes. Cardiovasc. Diabetol..

[29] Bansal N., Katz R., Seliger S., deFilippi C., Wettersten N., Zelnick L.R., Berry J.D., de Lemos J.A., Christenson R., Killeen A.A. (2022). Kidney Function Specific Reference Limits for N-terminal Pro Brain Natriuretic Peptide and High Sensitivity Troponin T: The Systolic Blood Pressure Intervention Trial. Kidney Med..

[30] Horii M., Matsumoto T., Uemura S., Sugawara Y., Takitsume A., Ueda T., Nakagawa H., Nishida T., Soeda T., Okayama S. (2013). Prognostic value of B-type natriuretic peptide and its amino-terminal proBNP fragment for cardiovascular events with stratification by renal function. J. Cardiol..

[31] Schaub J.A., Coca S.G., Moledina D.G., Gentry M., Testani J.M., Parikh C.R. (2015). Amino-Terminal Pro-B-Type Natriuretic Peptide for Diagnosis and Prognosis in Patients with Renal Dysfunction: A Systematic Review and Meta-Analysis. JACC Heart Fail..

[32] Fu S., Luo L., Ye P., Yi S., Liu Y., Zhu B., Wang L., Xiao T., Bai Y. (2013). The ability of NT-proBNP to detect chronic heart failure and predict all-cause mortality is higher in elderly Chinese coronary artery disease patients with chronic kidney disease. Clin. Interv. Aging.

[33] Navaneethan S.D., Roy J., Tao K., Brecklin C.S., Chen J., Deo R., Flack J.M., Ojo A.O., Plappert T.J., Raj D.S. (2016). Prevalence, Predictors, and Outcomes of Pulmonary Hypertension in CKD. J. Am. Soc. Nephrol..

[34] Yigla M., Fruchter O., Aharonson D., Yanay N., Reisner S.A., Lewin M. (2009). Pulmonary hypertension is an independent predictor of mortality in hemodialysis patients. Kidney Int..

[35] Vrabie A.M., Totolici S., Delcea C., Badila E. (2024). Biomarkers in Heart Failure with Preserved Ejection Fraction: A Perpetually Evolving Frontier. J. Clin. Med..

[36] Delcea C., Buzea C.A., Dobrev D., Dan G.A. (2024). Prognostic roles of neutrophil–lymphocyte, monocyte-lymphocyte and platelet-lymphocyte ratios for long-term all-cause mortality in heart failure. IJC Heart Vasc..

[37] Turi V., Sosdeanet R., Moleriu L., Damian G., Stoichescu-Hogeaal G., Iurciuc S., Dragan S. (2020). Correlations between left ventricle ejection fraction, global longitudinal strain by two-dimensional speckle tracking and pulse wave velocity in coronary artery disease. Med. Evol..

[38] Canepa M., Fonseca C., Chioncel O., Laroche C., Crespo-Leiro M.G., Coats A.J.S., Mebazaa A., Piepoli M.F., Tavazzi L., Maggioni A.P. (2018). Performance of Prognostic Risk Scores in Chronic Heart Failure Patients Enrolled in the European Society of Cardiology Heart Failure Long-Term Registry. JACC Heart Fail..

[39] Ivănescu A.C., Dan G.-A. (2021). Stroke risk scores to predict hospitalization for acute decompensated heart failure in atrial fibrillation patients. Rom. J. Intern. Med..

[40] Codina P., Lupón J., Borrellas A., Spitaleri G., Cediel G., Domingo M., Simpson J., Levy W.C., Santiago-Vacas E., Zamora E. (2021). Head-to-head comparison of contemporary heart failure risk scores. Eur. J. Heart Fail..

[41] Wada H., Shinozaki T., Suzuki M., Sakagami S., Ajiro Y., Funada J., Matsuda M., Shimizu M., Takenaka T., Morita Y. (2022). Impact of Chronic Kidney Disease on the Associations of Cardiovascular Biomarkers with Adverse Outcomes in Patients with Suspected or Known Coronary Artery Disease: The EXCEED-J Study. J. Am. Heart Assoc..

[42] Aletras G., Bachlitzanaki M., Stratinaki M., Lamprogiannakis E., Panagoutsos S., Kantartzi K., Georgopoulou T., Petrakis I., Foukarakis E., Pantazis Y. (2025). Unraveling Acute Cardiorenal Syndrome: Predictors and Consequences in Acute Heart Failure. J. Clin. Med..

[43] Lauer M.S., Blackstone E.H., Young J.B., Topol E.J. (1999). Cause of death in clinical research: Time for a reassessment?. J. Am. Coll. Cardiol..

[44] Morrow D.A., Wiviott S.D. (2019). Classification of Deaths in Cardiovascular Outcomes Trials Circulation. Circulation.

