# Supplementary material for: NT-proBNP as an Independent Predictor of Long-Term All-Cause Mortality in Heart Failure Across the Spectrum of Glomerular Filtration Rate
Source: J Clin Med. 2025 May 31;14(11):3886. doi: 10.3390/jcm14113886 (PMC12155938; doi:10.3390/jcm14113886)
Supplement: Supplementary file 1 [file jcm-14-03886-s001.zip › jcm-3624465-supplementary.pdf]

Supplementary Table S1. General clinical and laboratory characteristics of the study cohort on admission, across the eGFR subgroups.

		eGFR1<br>eGFR > 60<br>mL/min/1.73m² N = 471	eGRF2<br>eGFR 30 – 60<br>mL/min/1.73m²<br>N = 211	eGFR3<br>eGFR < 30<br>mL/min/1.73m²<br>N = 34	p value
**General characteristics**					
Age (years)		69.87 ± 10.02	75.22 ± 8.84	76.05 ± 10.42	<0.001
Gender (male)		244 (51.80%)	92 (43.60%)	11 (32.35%)	0.01
**Clinical characteristics**					
Heart rate (bpm)		79.94 ± 22.60	80.14 ± 24.02	76.84 ± 19.18	0.73
Systolic blood pressure (mmHg)		135.83 ± 23.34	136.11 ± 24.27	142.57 ± 30.90	0.44
Diastolic blood pressure (mmHg)		79.86 ± 11.88	78.35 ± 12.16	78.12 ± 15.89	0.26
**Heart failure characteristics**					
LVEF (%)		47.26 ± 12.97	45.83 ± 14.46	42.71 ± 14.52	0.11
HFpEF		353 (74.94%)	147 (69.66%)	21 (61.76%)	0.16
HFmrEF		53 (11.25%)	25 (11.85%)	7 (20.59%)	0.24
HFrEF		58 (12.31%)	38 (18.01%)	5 (14.71%)	0.14
NYHA I		26 (5.52%)	11 (5.21%)	2 (5.88%)	0.31
NYHA II		339 (71.97%)	132 (62.56%)	16 (47.06%)	
NYHA III		93 (19.74%)	62 (29.38%)	14 (41.18%)	
NYHA IV		11 (2.33%)	6 (2.84%)	1 (2.94%)	
Mitral regurgitation	Gr I	149 (31.63%)	61 (28.91%)	3 (8.82%)	0.34
	Gr II	175 (37.15%)	72 (34.12%)	17 (50.00%)	
	Gr III	61 (12.95%)	38 (18.01%)	4 (11.76%)	
	Gr IV	5 (1.06%)	3 (1.42%)	0	
Tricuspid regurgitation	Gr I	94 (19.95%)	38 (18.01%)	3 (8.82%)	0.58
	Gr II	95 (20.16%)	35 (16.58%)	6 (17.64%)	
	Gr III	23 (4.88%)	15 (7.11%)	3 (8.82%)	
	Gr IV	3 (0.63%)	3 (1.42%)	1 (2.94%)	
PASP (mmHg)		35.65 ± 14,62	40.11 ± 13.47	39.41 ± 14.73	0,002
IVC diameter (mm)		17.54±4.62	18.36 ± 4.26	19.40 ± 5.89	0.07
**Risk factors and comorbidities**					
Ischemic heart disease		197 (41.82%)	85 (40.28%)	19 (55.88%)	0.2
Prior myocardial infarction		73 (15.49%)	45 (21.33%)	11 (32.35%)	0.01
Stable angina		80 (16.98%)	35 (16.59%)	6 (17.64%)	0.9
HTN		400 (84.92%)	190 (90.05%)	29 (85.29%)	0.2
HTN grade	1	17 (3.61%)	6 (2.84%)	0	0.17
	2	123 (26.11%)	48 (22.75%)	4 (11.76%)	
	3	254 (53.93%)	128 (60.66%)	25 (73.53%)	
Diabetes mellitus		156 (33.12%)	77 (36.49%)	16 (47.06%)	0.22
Dyslipidemia		359 (76.22%)	165 (78.20%)	24 (70.59%)	0.6
History of stroke/ TIA		60 (12.74%)	27 (12.80%)	9 (26.47%)	0.07
AF		260 (55.20%)	135 (63.98%)	24 (70.59%)	0.03

Peripheral arterial disease	46 (9.76%)	15 (7.11%)	6 (17.64%)	0.1217
Obesity	178 (37.79%)	73 (34.60%)	13 (38.24%)	0.69
COPD	21 (4.45%)	16 (7.58%)	3 (8.82%)	0.18
**Laboratory parameters**				
Serum sodium (mmol/L)	140.55 ± 6.98	140.45 ± 3.42	137.12 ± 6.72	0.001
Serum potassium (mmol/L)	4.59 ± 4.29	4.54 ± 0.58	4.66 ± 0.61	<0.001
Serum chloride (mmol/L)	101.42 ± 5.68	101.45 ± 4.19	98.24 ± 7.60	0.01
Blood glucose (mg/dL)	116.21 ± 39.58	115.00 ± 33.85	142.58 ± 70.45	<0.001
Total cholesterol (mg/dL)	167.44 ± 48.05	159.23 ± 47.19	164.79 ± 48.19	0.13
Triglycerides (mg/dL)	102 (80.4-142.7)	114 (80.3-148.4)	138 (107.8-231)	0.005
HDL cholesterol (mg/dL)	49.72 ± 15.1	45.11 ± 12.34	42.01 ± 15.83	0.001
AST (UI/L)	22.00 (18.1-28.5)	21.30 (17.3-28.4)	23.35 (15.5-33.78)	0.6
ALT (UI/L)	20.80 (15.1-31)	17.85 (13.7-23.9)	18.55 (11.5-41.41)	0.009
NT-proBNP (pg/mL)	997 (461-2110)	1586 (871-3473)	4928 (2030-17464)	<0.001

AF atrial fibrillation ALT Alanine Aminotransferase AST Aspartate Aminotransferase Bpm beats per minute COPD Chronic Obstructive Pulmonary Disease eGFR Estimated Glomerular Filtration Rate HFpEF heart failure with preserved ejection fraction HFmEF Heart Failure with Mid-Range Ejection Fraction HFrEF Heart Failure with reduced Ejection Fraction LVEF Left Ventricular Ejection Fraction HTN Hypertension NYHA New York Heart Association TIA transient ischaemic attack
